# Use of health care services among people with Covid-19 symptoms in the first pandemic peak in France

**DOI:** 10.1371/journal.pone.0279538

**Published:** 2022-12-30

**Authors:** Jeanna-Eve Pousson, Léna Silberzan, Florence Jusot, Laurence Meyer, Josiane Warszawski, Nathalie Bajos, on behalf of the EpiCoV study group

**Affiliations:** 1 INSERM- IRIS, Aubervilliers, France; 2 Université Paris Dauphine, Paris, France; 3 CESP UMR 1018, Université Paris-Saclay, APHP, le Kremlin-Bicêtre, France; 4 EHESS, Aubervilliers, France; University of Oxford, UNITED KINGDOM

## Abstract

In France, the first pandemic peak fell disproportionately on the most disadvantaged, as they were overrepresented in contaminations and in developing severe forms of the virus. At that time, and especially during lockdown, the French healthcare system was severely disrupted and limited. The issue of social differences in the use of healthcare by people experiencing symptoms of Covid-19 arose. Based on a random sample of 135,000 persons, we selected respondents who reported Covid-19-like symptoms (cough, fever, dyspnea, anosmia and/or ageusia) during the first lockdown (n = 12,422). The aim of this study was to determine if the use of health care services was likely to contribute to widen Covid-19 social inequalities. Use of health care services was classified in three categories: (1) no consultation, (2) out-of-hospital consultation(s) and (3) in-hospital consultation(s). We estimated odds ratio of utilization of health care using multinomial regressions, adjusted on social factors (age, gender, class, ethno-racial status, social class, standard of living and education), contextual variables, health variables, and symptoms characteristics. Altogether, 37.8% of the individuals consulted a doctor for their symptoms; 32.1% outside hospital and 5.7% in hospital. Use of health care services was strongly associated with social position2: the most disadvantaged social groups and racially minoritized immigrants were more likely to use health care, particularly for in-hospital consultation(s). The highest utilization of health care were found among older adults (OR 9.51, 95%CI 5.02–18.0 compared to the youngest age group), the racially minoritized first-generation immigrants (OR 1.61, 95%CI 1.09–2.36 compared to the mainstream population), the poorest (OR 1.31, 95%CI 1.00–1.72) and the least educated (OR 2.20, 95%CI 1.44–3.38). To conclude, we found that the use of health care services counteracted the potential impact of social inequalities in exposure and infection to the Covid-19.

## Introduction

In France, like in many other countries, the first Covid-19 pandemic peak (March 2020—June 2020) revealed significant social inequalities in health [1–4]. The pandemic fell disproportionately on the most disadvantaged. Lower social class and racially minoritized groups were more exposed to contamination risks [5], and more often contracted severe forms of the virus [6], along with older individuals [7]. Thus, there is a need to analyze the medical care that these groups received when contracting the virus.

In France, during that period, access to care was deeply impacted. Indeed, the French system mostly relied on a containment strategy of the disease and social distancing in order to reduce new cases and delay the influx of acute patients in hospital. The most radical measure took place from March 17^th^ to May 10^th^, when a strict lockdown was implemented. Patients with Covid-19 symptoms were appealed to first contact a GP by phone, except for severe symptoms, in which case the recommendation was to call the French Emergency Medical Assistance Service. Furthermore, the French Health System was reorganized, resulting in a drastic health care rationing [8]. Health professionals activities decreased during that time period by 60% for specialists and 30% for GPs and midwives [9]. Moreover, doctors had to adapt their practices. The use of telemedicine was amplified: in April 2020, 27% of medical consultations were done with telemedicine, compared to less than 1% before the outbreak [10].

Access to care was not the only possible barrier to receiving medical attention when contracting Covid-19-like symptoms. Indeed, marked social inequalities in health care services utilization existed in France prior to the pandemic and had been well documented [11–13]. For the same health needs, ethno-racial minorities and people with lower education and income less frequently used health care than the rest of the population [14]. These inequalities were even more pronounced for care provided by specialists. A lower or later use in health care services could have contributed to worsen the impact of contracting the coronavirus, even more so for the most vulnerable.

There is a need to measure whether the French health system has been able to respond to this unprecedented health situation. There is also a need to clarify if overrepresented social groups in contaminations were also overrepresented in health care services utilization, when experiencing Covid-19-like symptoms. To our knowledge, this has not been done in the literature. However, two studies reported health care seeking due to Covid-19-like symptoms among older people, one in the US (n = 697) [15, 16] and one in Brazil (n = 568) [16]. While two different rates of people who sought care were found in these studies, 74.0% for the first and 33.6% for the second, neither of the studies found differences, according to race or to social factors, in seeking health care.

Using a large French population-based random survey, the aim of our study was to provide new insights on this topic by exploring the use of health care services of people with Covid-19-like symptoms.

## Participants and methods

### Population

The cohort was set-up in April 2020, with the general aim of understanding the main epidemiological, social and behavioral issues related to the Covid-19 epidemic in France [17]. The data collection period ran from May 2nd to June 2nd, 2020. In France, strict lockdown expanded from March 17th to May 10th.

A random sample of 350,000 people aged 15 and over was drawn from the tax database of the National Institute of Statistics and Economic Studies (INSEE), which covers 96% of the population living in France but excludes people living in institutional settings, and in particular older people living in collectivities. People who belonged to the lowest decile of income were over-represented. All those selected were sent a letter to participate in the survey. A total of 134,391 (38.4%) participated in the survey. Individuals were invited to answer the questionnaire online, or by phone for those who did not have Internet access. Furthermore, a random sample of 10% of people with Internet access was interviewed by phone in order to take into account a method collection effect.

Altogether, 134,391 individuals (38.4%) responded to the survey between May and June 2020, either online (82.3%) or by phone (17.7%). Reweighting, marginal calibrations and sampling design were used to correct for non-participation bias.

This study focused on adult participants (aged 18 and over) living in metropolitan France and who self-reported a sudden onset of at least one Covid-19-like symptom [18] among a cough, a fever, a dyspnea, anosmia, ageusia, or dysgeusia (n = 19,395). These symptoms were the most suggestive of Covid-19 at the time of the survey. We excluded individuals whose symptoms started before lockdown disruption in access to health care was mainly a consequence of lockdown (n = 5641, 29.1%). Data with missing values were also excluded (n = 1332, 9.7%). Altogether, 12,422 individuals were included in our study.

### Outcome

Participants who self-reported at least one Covid-19-like symptom provided information on their resulting health care services utilization. They reported if they had at least one medical consultation with a doctor either out of hospital (by phone, video, at home, in cabinet) or in hospital (emergency or not) since the beginning of lockdown. Our outcome was a three-category variable: (1) no consultation, (2) out-of-hospital consultation(s), and (3) in-hospital consultation(s). Those who both had consultations in-hospital consultation(s) and out-of-hospital consultation(s) were classified in (3) in-hospital consultation(s).

### Socio-demographic variables

We considered the following variables: age, sex, social class (based on current or last occupation), ethno-racial status (based on migration history), standard of living (based on decile of income per household consumption unit), formal education (defined according to the hierarchical grid of diplomas in France) and household composition. The ethno-racial status distinguished mainstream population, *i*.*e*. persons residing in metropolitan France who are neither immigrants nor native to French Overseas Departments (FOD, *i*.*e*. Martinique, Guadeloupe, Reunion Island), nor descendants of immigrant(s) or of FOD native [5]. For the racially minoritized groups (Milner 2020), a distinction was made according to the first (immigrants) and second (descendants of immigrants) generations of immigration and the country of origin. The term “racially minoritized” refers to people from the Maghreb, Turkey, Asia and Africa.

### Health variables

Health variables reflected health care needs in general, risk factors related to a Covid-19 severe infection, and the severity of the related infection. They included the perceived health status, the body mass index (BMI), and the report of chronic diseases.

### Covid-19-like symptoms characteristics

Strength and severity of the Covid-19-like symptoms (a cough, a fever, a dyspnea, anosmia, ageusia, or dysgeusia) were measured with the following variables: number of symptoms and duration of symptoms. We also considered the number of other symptoms during lockdown possibly linked to the virus, which can reflect a necessity of care (headache, unusual fatigue, muscle soreness, breathing difficulties, unusual shortness of breath, a runny nose, nausea, vomiting, diarrhea, chest pain, appearance of rashes).

### Other covariates

The three most affected regions at the time of the survey (Grand Est, Hauts-de-France, Ile-de-France) were distinguished to account for regional variations in incidence of Covid-19 infections. We added the Local Potential Accessibility (LPA) indicator, an indicator that measures accessibility to private GPs, using the doctor activity level, the population’s health care needs and the health care supply in the surrounding communes. The higher the score is, the more access to private GPs the location offers. Being part of the essential workforce (including healthcare workers) and working outside the place of residence during lockdown were also considered as they may have increased accessibility of medical consultation(s) and may have required a medical leave delivered by healthcare professionals at the onset of symptom(s). Confounding variables included the self-reported belief that the Covid-19-like symptoms were the consequence of being infected with the coronavirus.

### Statistical analyses

Use of health care services was first described according to the respondents’ demographic, socio-economic (Table 1), and health characteristics (Table 2). In order to test the association between use of health care services and social factors, adjusted odds ratios (ORs) on all variables presented in Tables 1 and 2. were assessed by multinomial logistic regression. All analyses were weighted using a Heckman model to take into account the effect of the response mode on the reporting of Covid-19-like symptoms. The threshold value for *p* was <0.05. Statistical analyses were performed using SAS 9.4.

**Table 1:** Distribution of the use of health care services by socio-demographic characteristics.

	No consultation n = 7,799 (62.2%)	Out-of-hospital consultation(s) n = 3,966 (32.1%)	In-hospital consultation(s) *n = 657 (5*.*7%)*	Total n = 12,422 (100%)
**Sex**				
Men	3,510 (66.0)	1,530 (28.8)	247 (5.2)	5,287 (43.9)
Women	4,289 (59.2)	2,436 (34.7)	410 (6.0)	7,135 (56.1)
**Age**				
18–24	1,143 (73.1)	361 (24.4)	43 (2.5)	1,547 (12.1)
25–34	1,504 (65.3)	714 (30.0)	116 (4.7)	2,334 (20.8)
35–44	1,785 (62.0)	928 (32.6)	142 (5.4)	2,855 (23.0)
45–54	1,653 (60.5)	910 (33.5)	159 (6.0)	2,722 (20.4)
55–64	1,071 (57.5)	636 (36.0)	99 (6.5)	1,806 (13.4)
65–74	505 (56.2)	315 (36.4)	59 (7.4)	879 (6.9)
75 and over	138 (46.6)	102 (37.8)	39 (15.6)	279 (3.4)
**Ethno-racial status**				
Mainstream population	6,201 (63.0)	3,086 (31.8)	493 (5.2)	9,780 (76.7)
Born or parents born in FOD	136 (57.5)	77 (36.0)	13 (6.5)	226 (1.9)
Non-racially minoritized second-generation immigrants	442 (64.6)	225 (30.4)	30 (5.1)	697 (5.7)
Racially minoritized second-generation immigrants	433 (60.8)	225 (32.5)	43 (6.7)	701 (6.1)
Non-racially minoritized first-generation immigrants	234 (53.1)	155 (39.3)	23 (7.6)	412 (3.8)
Racially minoritized first-generation immigrants	353 (57.8)	198 (31.9)	55 (10.4)	606 (5.9)
**Social class**				
Self-employed and entrepreneurs	380 (61.5)	179 (32.3)	35 (6.2)	594 (4.8)
Senior executive professionals	1,941 (65.7)	924 (30.5)	109 (3.8)	2,974 (21.9)
Middle executive professionals	1,528 (61.6)	814 (32.8)	140 (5.6)	2,482 (19.6)
Employees	1,855 (58.3)	1,104 (35.3)	201 (6.5)	3,160 (26.3)
Manual workers	744 (61.6)	387 (31.3)	72 (7.1)	1,203 (11.1)
Never worked and others	1,351 (65.1)	558 (28.9)	100 (6.0)	2,009 (16.4)
**Standard of living (in deciles)**				
D1-D3 (lowest)	1,859 (61.5)	910 (31.3)	189 (7.2)	2,958 (24.5)
D4-D7	2,768 (59.9)	1,549 (33.9)	267 (6.2)	4,584 (39.5)
D8-D10	3,172 (65.3)	1,507 (30.7)	201 (4.0)	4,880 (36.0)
**Formal education**				
No diploma or primary education	664 (54.7)	387 (34.2)	110 (11.1)	1,161 (13.1)
Vocational secondary	1,110 (55.2)	759 (37.7)	141 (7.1)	2,010 (16.4)
High school	1,824 (63.7)	891 (31.6)	132 (4.7)	2,847 (22.7)
High school +2 to +4 years	2,593 (64.6)	1,246 (30.8)	188 (4.6)	4,027 (30.4)
High school +5 or more years	1,608 (68.2)	683 (28.3)	86 (3.5)	2,377 (17.5)
**Household composition**				
Lives alone	1,105 (63.1)	506 (30.3)	89 (6.6)	1,700 (14.7)
Lives with a least a child of a teenager	2,922 (60.6)	1,595 (33.7)	264 (5.6)	4,781 (38.0)
Other compositions	3,772 (63.2)	1,865 (31.4)	304 (5.4)	5,941 (47.3)
**Work situation**				
Did not work	3,042 (62.8)	1,471 (30.9)	275 (6.4)	4,788 (39.8)
Only worked remotely	1,550 (68.6)	649 (28.1)	74 (3.3)	2,273 (17.0)
Worked outside the place of residence	3,207 (59.2)	1,846 (34.9)	308 (6.0)	5,361 (43.2)
**Part of the essential workforce**				
No	5,145 (64.0)	2,417 (30.6)	382 (5.3)	7,944 (64.3)
Yes, healthcare workers	395 (50.6)	286 (37.3)	100 (12.1)	781 (5.9)
Yes, other	2,259 (60.6)	1,263 (34.3)	175 (5.1)	3,697 (29.8)
**Region of residence according to Covid-19 infections at the time of the survey**				
Least affected regions	4,303 (62.7)	2,171 (32.3)	332 (5.0)	6,806 (55.4)
Grand Est	876 (58.9)	512 (35.3)	75 (5.8)	1,463 (10.0)
Hauts-de-France	806 (62.5)	400 (30.7)	88 (6.9)	1,294 (9.6)
Ile-de-France	1,814 (62.2)	883 (31.1)	162 (6.7)	2,859 (25.0)
**Local Potential Accessibility (LPA)**				
1	823 (64.1)	375 (29.9)	72 (6.0)	1,270 (9.5)
2	5,511 (62.1)	2,822 (32.3)	463 (5.6)	8,796 (71.2)
3	1,465 (61.8)	769 (32.5)	122 (5.8)	2,356 (19.3)

**Table 2:** Distribution of the use of health care services by health characteristics.

	No consultation n = 7,799 (62.2%)	Out-of-hospital consultation(s) n = 3,966 (32.1%)	In-hospital consultation(s) *n = 657 (5*.*7%)*	Total n = 12,422 (100%)
**Asthma or other respiratory diseases**				
No	7,184 (62.9)	3,545 (31.6)	567 (5.5)	11,296 (90.7)
Yes	615 (54.9)	421 (37.4)	90 (7.8)	1,126 (9.3)
**Diabetes, cancer or HIV**				
No	7,407 (63.0)	3,679 (31.7)	586 (5.2)	11,672 (93.6)
Yes	392 (50.0)	287 (38.0)	71 (12.0)	750 (6.4)
**Other chronic diseases or functional limitations**				
No	6,450 (62.9)	3,187 (31.6)	534 (5.6)	10,171 (81.8)
Yes	1,349 (59.2)	779 (34.6)	123 (6.2)	2,251 (18.2)
**Hypertension**				
No	7,225 (63.2)	3,568 (31.3)	580 (5.5)	11,373 (91.1)
Yes	574 (52.2)	398 (40.1)	77 (7.7)	1,049 (8.9)
**Cardiac diseases**				
No	7,617 (62.6)	3,841 (32.0)	622 (5.4)	12,080 (97.0)
Yes	182 (49.5)	125 (36.9)	35 (13.6)	342 (3.0)
**BMI**				
Underweight	298 (61.2)	162 (33.2)	25 (5.6)	485 (3.9)
Normal	4,222 (65.2)	1,921 (30.4)	280 (4.5)	6,423 (51.2)
Overweight	2,197 (59.7)	1,230 (33.5)	220 (6.7)	3,647 (29.7)
Obesity	1,082 (57.4)	653 (35.0)	132 (7.7)	1,867 (15.3)
**Perceived health status**				
Good	7,547 (63.0)	3,760 (31.8)	591 (5.2)	11,898 (95.3)
Bad	252 (46.1)	206 (38.6)	66 (15.3)	524 (4.7)
**Number of Covid-like symptoms**				
1	5,519 (72.1)	1,810 (24.3)	243 (3.6)	7,572 (61.4)
2	1,667 (55.0)	1,170 (38.9)	179 (6.2)	3,016 (24.1)
3	522 (36.8)	707 (52.8)	135 (10.4)	1,364 (10.8)
4	91 (19.6)	279 (57.5)	100 (22.9)	470 (3.7)
**Number of other symptoms**				
0	1,585 (72.9)	469 (23.6)	68 (3.5)	2,122 (17.2)
1	4,767 (67.5)	2,001 (28.3)	277 (4.3)	7,045 (57.1)
2	1,447 (43.4)	1,496 (46.4)	312 (10.3)	3,255 (25.7)
**Onset of anosmia or agueunia during lockdown**				
No	6,795 (65.8)	2,989 (29.5)	445 (4.7)	10,229 (82.2)
Yes	1,004 (45.3)	977 (44.4)	212 (10.3)	2,193 (17.8)
**Onset of a fever during lockdown**				
No	3,827 (70.0)	1,354 (25.2)	231 (4.8)	5,412 (44.2)
Yes	3,972 (56.0)	2,612 (37.6)	426 (6.4)	7,010 (55.8)
**Onset of dyspnea during lockdown**				
No	5,969 (67.8)	2,477 (28.8)	273 (3.4)	8,719 (70.3)
Yes	1,830 (48.9)	1,489 (40.0)	384 (11.1)	3,703 (29.7)
**Did the symptoms last for more than a week?**				
No	3,656 (76.5)	985 (21.0)	113 (2.4)	4,754 (38.3)
Yes	4,143 (53.3)	2,981 (39.0)	544 (7.7)	7,668 (61.7)
**Self-reported belief that the Covid-like symptoms were the result of being infected with the coronavirus**				
No	3,429 (74.1)	980 (22.0)	153 (3.9)	4,562 (37.6)
Yes	4,370 (55.0)	2,986 (38.2)	504 (6.7)	7,860 (62.4)

### Ethics statement

The studies involving human participants were reviewed and approved by the CNIL (Commission nationale de l’informatique et des libertés, the French independent administrative authority responsible for data protection), the Comité de protection des personnes (French equivalent of the Research Ethics Committee), and the Comité du Label de la statistique publique. Written informed consent to participate in this study was provided by the participants’ legal guardian/next of kin.

## Results

In our sample, 37.8% of those who reported cough, fever, dyspnea, anosmia, ageusia and/or dysgeusia consulted a doctor for these symptoms; 32.1% outside hospital and 5.7% in hospital (Table 1). Women used health care services more than men with stronger differences for out-of-hospital use consultation(s) (34.7% *versus* 28.8%). There was a clear positive gradient between age and consultation(s) rates: the older individuals were, the more they consulted at least once, whether it be in-hospital consultation(s) (2.5% of the 18–24 years *versus* 15.6% of the 75 years or more) or out-of-hospital consultation(s) (24.4% *versus* 37.8% outside hospital). Differences in consultation(s) rates were also found according to the ethno-racial statuts. FOD or descendants of FOD natives and first-generation immigrants more often consulted doctors than the mainstream population. The highest in-hospital consultation(s) rate was found among racially minoritized first-generation immigrants (10.4% *versus* 5.2% of the mainstream population). Health care utilization was also strongly associated with social position. Senior executive professionals consulted doctors less often than manual workers (34.3% and 38.4% respectively), with marked differences regarding in-hospital consultation(s) (3.8% *versus* 7.1%). A similar trend was found regarding incomes with a lower in-hospital consultation(s) rate among the richest, compared to the poorest (4.0% *versus* 7.2%). Finally, there was a distinct negative gradient between in-hospital consultation(s) and education: the more educated the respondents were, the less they used in-hospital health care services (from 11.1% for those without any diploma to 3.5% for those with the highest education level).

When it comes to health variables, 62.4% of the respondents attributed the Covid-19-like symptoms to an infection to the coronavirus and the symptoms lasted for over than a week for 61.7% of the respondents (Table 2). Respondents who suffered from a chronic disease consulted a doctor more often than the rest of the population. The highest rates were observed among those who suffered from cardiac diseases (50.5% *versus* 37.8%). More generally, respondents who perceived their health as bad consulted more often (53.9% *versus* 37.0%), especially in hospitals (15.3% *versus* 5.2%).

Results from the multinomial analysis comparing use of health care services (in-hospital consultation(s), out-of-hospital consultation(s) *versus* no consultation) among those with Covid-19-like symptoms are presented in Table 3. After adjustments on health variables, characteristics of Covid-19-like symptoms and variables related to consultation(s) accessibility and needs of health care, results confirmed that women were more likely than men to consult a doctor, but only for out-of-hospital consultation(s) (OR, 95% CI: 1.25 (1.13–1.37)). Positive association between age and medical consultation(s) was found and was the strongest regarding in-hospital care (compared to the 18–24, up to 9.51 (5.02–18.0) for 75 and older for in-hospital care and 2.58 (1.73–3.84) for out-of-hospital care). Racially minoritized first-generation immigrants consulted doctors more often than the mainstream population, but for in-hospital care (1.61 (1.09–2.36)). While no association was found between social class and health care use, results showed a negative association with level of education: the more educated respondents were, the less consulted doctors, with larger gaps for in-hospital consultation(s). Compared to respondents with the highest education level, respondents with no diploma or a primary education level used in- and out-of-hospital care more often (respectively 1.34 (1.08–1.67) and 2.20 (1.44–3.38)). Similar associations were found with standard of living, with a greater use of consultation(s) rates among middle deciles (respectively 1.11 (1.00–1.24) and 1.38 (1.10–1.74), compared to the richest 30%).

**Table 3:** Factors associated with use of health care services (multinomial regression, reference = no consultation).

*Ref*.: *No consultation*	*Out-of-hospital consultation(s) OR (95% CI)*	*In-hospital consultation(s) OR (95% CI)*
**Sex**		
Men	*Ref*.	*Ref*.
Women	**1.25 (1.13–1.37)**	1.02 (0.82–1.27)
**Age**		
18–24	*Ref*.	*Ref*.
25–34	1.17 (0.96–1.44)	**1.66 (1.05–2.65)**
35–44	1.19 (0.96–1.47)	**1.81 (1.07–3.04)**
45–54	1.16 (0.94–1.43)	**1.78 (1.12–2.83)**
55–64	**1.44 (1.17–1.79)**	**2.25 (1.40–3.63)**
65–74	**1.79 (1.37–2.33)**	**3.59 (2.02–6.37)**
75 and over	**2.58 (1.73–3.84)**	**9.51 (5.02–18.0)**
**Ethno-racial status**		
Mainstream population	*Ref*.	*Ref*.
Born or parents born in FOD	1.41 (0.99–2.02)	1.40 (0.75–2.63)
Non-racially minoritized second-generation immigrants	0.90 (0.75–1.09)	0.91 (0.56–1.47)
Racially minoritized second-generation immigrants	1.16 (0.94–1.43)	1.41 (0.91–2.18)
Non-racially minoritized first-generation immigrants	1.28 (0.97–1.69)	0.99 (0.59–1.65)
Racially minoritized first-generation immigrants	1.05 (0.83–1.32)	**1.61 (1.09–2.36)**
**Social class**		
Self-employed and entrepreneurs	0.88 (0.69–1.12)	1.05 (0.64–1.72)
Senior executive professionals	*Ref*.	*Ref*.
Middle executive professionals	0.91 (0.78–1.05)	0.95 (0.68–1.33)
Employees	0.95 (0.82–1.11)	1.19 (0.85–1.66)
Manual workers	0.86 (0.70–1.05)	1.12 (0.72–1.74)
Never worked and others	0.99 (0.81–1.21)	1.41 (0.94–2.10)
**Standard of living (in deciles)**		
D1-D3 (lowest)	0.96 (0.84–1.11)	**1.31 (1.00–1.72)**
D4-D7	**1.11 (1.00–1.24)**	**1.38 (1.10–1.74)**
D8-D10	*Ref*.	*Ref*.
**Formal education**		
No diploma or primary education	**1.34 (1.08–1.67)**	**2.20 (1.44–3.38)**
Vocational secondary	**1.56 (1.30–1.88)**	**1.80 (1.20–2.68)**
High school	**1.17 (1.00–1.38)**	1.21 (0.84–1.75)
High school +2 to +4 years	1.11 (0.97–1.28)	1.19 (0.86–1.65)
High school +5 or more years	*Ref*.	*Ref*.

Also adjusted on region of main residence, LPA (Local Potential Accessibility), household composition, work situation, being part of the essential workforce, chronic diseases, self-perceived health status, BMI, nature, quantity, and duration of Covid-19-like symptoms, number of other symptoms, self-belief that the Covid-19-like symptoms were the consequence of being infected with the coronavirus.

## Discussion

To our knowledge, this study is the first to describe use of health care services among people aged 18 and over with Covid-19-like symptoms during the first wave of the epidemic. Regardless of their health status and the severity of their Covid-19 related symptoms, we found that women, older adults, disadvantaged social groups and racialized first-generation immigrants were significantly more likely to use health care services, in particular in-hospital care, than the rest of the population.

The health care utilization patterns observed during our study duplicated those before Covid-19. They may also have been reinforced by the Covid-19 peak context. Indeed, women being both recipients and providers of healthcare [19], they are used to visiting GPs more often than men [20]. Pre-Covid-19 evidence shows that older adults consulted doctors (in- and out-of-hospital) more often than younger adults [21]. Older adults were also particularly targeted by prevention campaigns during the first pandemic peak, which contributes to explaining why for the same symptom severity, they used health care services more often. The most socially disadvantaged are known to use GP services and to consult doctors in hospitals more often than the rest of the population. An explanation lies in the nature of the French Health system that guarantees an extensive coverage of health expenditures. Furthermore, in a study on over 3,000 GPs, 1 GP out of 4 reported initiatives to contact patients during lockdown with a strategy including social criteria [22]. Similar pre-Covid-19 patterns were also found according to ethno-racial status [23–25]. Immigrants’ higher in-hospital consultations rate could be partly explained by a lower use of telemedicine. A study among NYC patients showed that difficulties in the utilization of digital healthcare among the ethnic minorities continued to be observed during the early phase of the pandemic [26]. Contrary to the most disadvantaged and ethno-racial minorities, the most advantaged and the mainstream population refer to specialists to a greater account. It is noteworthy that during the first Covid-19 peak, specialists of the disease did not yet exist. As a matter of fact, nearly all the options to receive medical care were in-hospital and GPs could be consulted for expert advice.

Very few social differences in health care utilization during the first Covid-19 peak when presenting Covid-19-like symptoms were found. Only two studies looked into rates of health care seeking among older people with Covid-19-like symptoms by race in the US [15] and social factors in the US and in Brazil [15, 16]. Although the studies did not take place during lockdown, they were still conducted at a time of a peak of the pandemic and great disruption to the Health systems. In the Brazilian study (n = 568), authors found that 33.6% of people aged 50 or over sought care for Covid-19 related symptoms. This rate reached 74.0% in the US study (n = 697), where the average age was 76.8 years. In our study, 45.0% of people aged 50 and over had at least one medical consultation and the rate rose to 53.4% for those aged 75 and over. In both studies, no health inequalities regarding health care seeking was found, according to race (US study), sex, age (Brazilian study), income and education (both studies). When we restricted our analyses to people aged 50 and over (n = 4177, S1 Table), differences still persisted according to social factors: the older and the less educated respondents were, the more they used in-hospital health care. Conflicting results with the two previous studies may result from several factors. First, we studied health use of health care services and not health care seeking. Although in our study, among people who did not consult a doctor, only 1.0% did not due to barriers to health care access (no nearby doctor, long delays to get doctor’s appointment, financial costs). Moreover, sensitive analyses in the Brazilian study suggest that barriers to health care access were not associated with care seeking. Due to fewer included individuals, previous studies may suffer from lack of statistical power.

The social differences that our study found in health care services utilization might be in some measure explained by other factors, such as the fear of the disease. The poorest and the least educated have been shown to have higher levels of Covid-19 fear [27–29], which could potentially lead them to consult a doctor or go to the hospital more often than their counterparts when presenting Covid-19-like symptoms. Compared to the mainstream population, racialized minorities accumulated more exposure factors during the pandemic peak [5], and lived more often in overcrowded households [5], which could have fueled the feeling of fear of the virus.

Our study shows limitations. First, as any national population-based survey, the study fails to capture highly vulnerable groups such as undocumented migrants and homeless people, who are particularly affected by the pandemic [30]. Additionally, it is possible that the most severe cases have been under-represented in our study, which may relate to the most socially disadvantaged and ethno-racial minorities more [1]. Secondly, participants were questioned on their use of health care services for at least one of 12 items list of symptoms suggestive of Covid-19 while we have retained only five. It is therefore possible that some participants reported use of health care services for other symptom(s) than those selected.

Several sensitivity analyses were done. We excluded individuals whose symptoms appeared in the week preceding their response to the questionnaire, as they had a shorter period of time to visit a doctor than others. We then adjusted our final model on the fear of contracting Covid-19 when consulting a doctor. In both cases results and conclusions remained unchanged. As we studied health care access, people who sought care and did not have any medical consultation could not be identified. However, in another question, we asked participants what they first did at the onset of the first symptom: among those who did not use health care services during the whole study period, less than 2% unsuccessfully sought care. Therefore, one might think that a very low percent of people who did use health services at the onset of the symptom unsuccessfully sought care after. Finally, in our analyses, the region variable was included as a control variable even though it is a contextual variable. We therefore calculated clustered sandwich estimators, which are used to adjust inference when errors are correlated within clusters (e.g. region). Our results were very similar, to the nearest hundredth, to those we obtained when we simply adjust on region. This means that biases resulting from not taking the region variable as a cluster seem to be minors.

In conclusion, our results seem to reflect pre-existing social differences in health care utilization patterns. Those patterns might have been strengthened by the context of the Covid-19 peak, and the resulting fear of the virus. In fact, social groups who were the most at risk were also those who have consulted the most. As a consequence, the potential impact of social inequalities in exposure and infection to the Covid-19 [5] seemed not to have been widened by the use of a free health care system.

## Supporting information

S1 Table Factors associated with use of health care services among people aged 50 and over, n = 4177 (multinomial regression, reference = no consultation). Also adjusted on region of main residence, LPA (Local Potential Accessibility), household composition, work situation, being part of the essential workforce, chronic diseases, self-perceived health status, BMI, nature, quantity, and duration of Covid-19-like symptoms, number of other symptoms, self-belief that the Covid-19-like symptoms were the consequence of being infected with the coronavirus.
(PDF)
