# Supplementary material for: Use of health care services among people with Covid-19 symptoms in the first pandemic peak in France
Source: PLoS One. 2022 Dec 30;17(12):e0279538. doi: 10.1371/journal.pone.0279538 (PMC9803216; doi:10.1371/journal.pone.0279538)
Supplement: S1 Table — Also adjusted on region of main residence, LPA (Local Potential Accessibility), household composition, work situation, being part of the essential workforce, chronic diseases, self-perceived health status, BMI, nature, quantity, and duration of Covid-19-like symptoms, number of other symptoms, self-belief that the Covid-19-like symptoms were the consequence of being infected with the coronavirus. (PDF) [file pone.0279538.s001.pdf]

S1 Table. Factors associated with use of health care services among people aged 50 and over, n=4177 (multinomial regression, reference = no consultation).

Ref.: No consultation	*Out-of-hospital consultation(s) OR (95% CI)*	*In-hospital consultation(s) OR (95% CI)*
**Sex**		
Men	*Ref.*	*Ref.*
Women	1.05 (0.88-1.25)	0.89 (0.62-1.30)
**Age**		
50-54	*Ref.*	*Ref.*
55-64	**1.21 (1.00-1.47)**	**1.55 (1.00-2.40)**
65-74	**1.33 (1.00-1.76)**	**2.54 (1.38-4.66)**
75 and over	**1.85 (1.23-2.79)**	**6.31 (3.07-13.0)**
**Ethno-racial status**		
Mainstream population	*Ref.*	*Ref.*
Born or parents born in FOD	1.11 (0.70-1.76)	0.81 (0.40-1.61)
Non-racially minoritized second-generation immigrants	0.92 (0.67-1.26)	0.80 (0.39-1.64)
Racially minoritized second-generation immigrants	1.18 (0.77-1.80)	1.05 (0.52-2.13)
Non-racially minoritized first-generation immigrants	1.29 (0.77-2.16)	1.76 (0.65-4.79)
Racially minoritized first-generation immigrants	1.01 (0.47-2.16)	1.24 (0.37-4.10)
**Social class**		
Self-employed and entrepreneurs	0.83 (0.58-1.19)	0.82 (0.41-1.64)
Senior executive professionals	*Ref.*	*Ref.*
Middle executive professionals	0.99 (0.78-1.26)	0.63 (0.36-1.11)
Employees	0.90 (0.70-1.16)	1.08 (0.64-1.83)
Manual workers	0.75 (0.53-1.06)	0.98 (0.48-1.99)
Never worked and others	1.03 (0.74-1.44)	1.26 (0.67-2.37)
**Standard of living (in deciles)**		
D1-D3 (lowest)	1.07 (0.83-1.38)	0.98 (0.62-1.53)
D4-D7	1.07 (0.88-1.29)	1.03 (0.71-1.48)
D8-D10	*Ref.*	*Ref.*
**Formal education**		
No diploma or primary education	1.36 (0.98-1.89)	3.26 (1.66-6.43)
Vocational secondary	1.52 (1.13-2.05)	2.62 (1.33-5.16)
High school	1.10 (0.82-1.47)	2.08 (1.08-4.02)
High school +2 to +4 years	1.03 (0.80-1.32)	1.36 (0.72-2.54)
High school +5 or more years	*Ref.*	*Ref.*

Also adjusted on region of main residence, LPA (Local Potential Accessibility), household composition, work situation, being part of the essential workforce, chronic diseases, self-

perceived health status, BMI, nature, quantity, and duration of Covid-19-like symptoms, number of other symptoms, self-belief that the Covid-19-like symptoms were the consequence of being infected with the coronavirus.
